# Yield Load Solutions for SE(B) Fracture Toughness Specimen with I-Shaped Heterogeneous Weld

**DOI:** 10.3390/ma15010214

**Published:** 2021-12-28

**Authors:** Pejo Konjatić, Marko Katinić, Dražan Kozak, Nenad Gubeljak

**Affiliations:** 1 Mechanical Engineering Faculty, University of Slavonski Brod, Trg Ivane Brlic Mazuranic 2, 35000 Slavonski Brod, Croatia; mkatinic@unisb.hr (M.K.); dkozak@unisb.hr (D.K.); 2 Faculty of Mechanical Engineering, University of Maribor, Smetanova 17, SI-2000 Maribor, Slovenia; nenad.gubeljak@um.si

**Keywords:** yield load, heterogeneous weld, numerical analysis, SE(B) specimen

## Abstract

The objective of this work was to investigate the fracture behavior of a heterogeneous I-shaped welded joint in the context of yield load solutions. The weld was divided into two equal parts, using the metal with the higher yield strength and the metal with the lower yield strength compared to base metal. For both configurations of the I-shaped weld, one with a crack in strength in the over-matched part of the weld and one for a crack in the under-matched part of the weld, a systematic study of fracture toughness SE(B) specimen was carried out in which the crack length, the width of the weld and the strength mismatch factor for both weld metals were varied, and the yield loads were determined. As a result of the study, two mathematical models for determination of yield loads are proposed. Both models were experimentally tested with one strength mismatch configuration, and the results showed good agreement and sufficiently conservative results compared to the experimental results.

## 1. Introduction

Joining metals by welding is nowadays widely used in the construction of most engineering structures. The requirements for high quality welded joints joining similar or dissimilar metals, taking into account the mechanical properties of the metal, lead to the production of welded joints with significant differences in strength compared to the base metal.

Like all structures, welded structures are susceptible to damage during use, particularly in the weld or heat-affected zone, due to the change in metal properties and the expected significant nonlinear deformations caused by mechanical heterogeneity. Repaired welds are commonly used in steel structures either to correct initial fabrication defects or to repair damage during service to extend the service life of the structure [1]. When welds are repaired, additional heterogeneity is introduced into the already heterogeneous structure.

In the conventional evaluation of the safe operation of defect-free structures, the applied stresses are compared to a limit stress, such as the yield strength of the material. When damage in the form of a crack is present, the assessment of welded joints is based on the evaluation of the stress intensity factor, the J-integral and the crack tip opening displacement (CTOD) [2]. On the other hand, the influence of mechanical heterogeneity on the fracture behavior of welds is not explicitly included in the mentioned fracture mechanics parameters. However, methods and procedures for evaluating homogeneous and heterogeneous structures, which have been developed recently, can be used to determine whether or not the structure is safe for further exploitation. 

One commonly used procedure for a structural integrity assessment is the SINTAP procedure (Structural INTegrity Assessment Procedure) [3]. The application of the SINTAP procedure is based on the implementation of the yield load solution in the failure assessment diagram (FAD) to determine the safe operation of the assessed structure. There are a number of studies dealing with various aspects of the fracture behavior of homogeneous welds with strength mismatch compared to the base metal, including recent ones [4,5,6,7,8,9], as well as a number of studies dealing with heterogeneity in welds with strength mismatch [10,11,12,13,14,15,16].

A common parameter for describing the level of strength mismatch between individual metals, in the context of this investigation, between base metal and weld metals, i.e., mismatch in yield strength between the weld metal and the base metal, is quantified by the mismatch factor *M*:

(1) $$M=\frac{\sigma_{\mathrm{YW}}}{\sigma_{\mathrm{YB}}}$$

where *σ*_YW_ and *σ*_YB_ represent the yield strength of the weld metal and the yield strength of the base metal (BM), respectively, while *M* < 1 refers to under-matching (UM) and *M* > 1 to over-matching (OM). 

Yield load solutions are available for a limited number of strength mismatch configurations for over-matched and under-matched welds [17,18,19,20], but only very limited and partial solutions in situations where additional heterogeneity due to repair weld metal is present in another level of the strength mismatch [21,22]. Therefore, this research aims to extend the existing yield load solutions to I-shaped heterogeneous weld solutions in order to gain insight into the fracture behavior of the repaired weld and open the possibility of applying structural assessment procedures for repair welds. As a result, a compendium of yield load solutions for a standard fracture mechanics specimen SE(B) with a heterogeneous weld is given, which can be used as the input parameter for an assessment using standard structural integrity assessment procedures. 

## 2. Problem Description and Investigation Plan

Since butt welds are used extensively in the welding industry, there is often a need to repair such welds when defects occur during welding or during the service life of the welded structure. If the repair involves the use of a filler metal different from the filler metal used to weld the original weld, the result is a heterogeneous welded joint with two different weld metals in addition to the base metal. When structures are put back into service after repair, the occurrence of cracks in the original part or in the repaired part of the weld is possible again.

For this reason, and for the reasons given in the introduction, a study of the fracture behavior of an I-shaped butt weld was carried out. The effects of weld damage in the form of a crack were analyzed.

**Figure 1:**
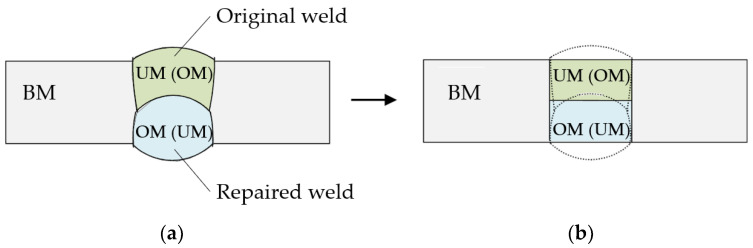
Idealization of repaired I-shaped butt-welded joint: (**a**) heterogeneous welded joint; (**b**) idealized heterogeneous welded joint.

Due to the complexity of the problem to be analyzed, it was necessary to introduce certain idealizations and simplifications. In all previous studies on a similar topic, several such idealizations were introduced, starting from the weld geometry idealized by a rectangle, and the observed cracks were located at the interface of dissimilar materials or in the middle of the weld due to the nature of crack formation described in [18,23,24]. In this study, the I-shaped weld was also idealized as a rectangular shape, as well as the original and repaired part of the weld (Figure 1), and the crack was located in the center of the weld. In [25], researchers have demonstrated that the mechanical properties of the heat-affected zone have a negligible effect on the stress concentration at the crack tip when the crack tip is located in the center of the weld. However, if the crack tip is located in or near the heat-affected zone, the properties of the heat-affected zone have to be taken necessarily into account [26]. Since the crack in the middle of the weld was analyzed here, the heat-affected zone was omitted. 

The difference in elastic properties of the material as well as the strain hardening of the material affect the fracture behavior of the weld, but here, only the influence of the degree of strength mismatch between the single welded metal and the base metal is studied. In addition to the strength mismatch, the change in weld width and the crack size were also analyzed. The influence of mentioned geometrical and mechanical parameters on the yield load was observed, i.e., the load at which the metal flows through the entire cross-section of the weld, since at that moment a plastic hinge is formed.

## 3. Finite Element Analysis

In order to investigate the influence of weld material heterogeneity on weld fracture behavior, the weld area was divided into two zones of equal size but consisted of different metals. The first zone represented the original weld before repair, while the second zone represented the repaired portion of the weld. In the first variant, the zone of the original weld was made of a metal whose yield strength was lower than the yield strength of the base metal (UM), while the second half of the weld was made of a metal whose yield strength was higher than the yield strength of the base metal (OM). In the second variant, positions of the UM and OM part of the weld were reversed. Combinations where both weld metals have over-match or under-match character were not covered by this investigation. 

**Figure 2:**
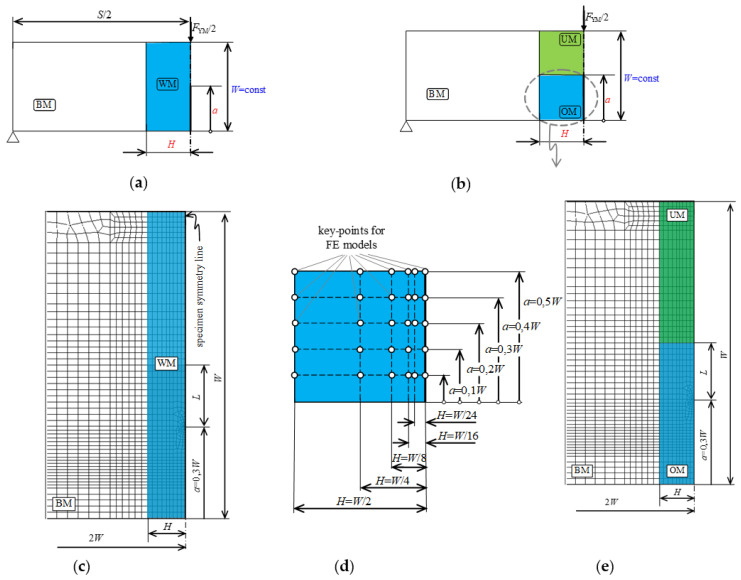
Numerical model: (**a**) homogeneous weld for verification; (**b**) heterogeneous weld; (**c**) detail of finite element mesh of homogeneous weld for verification; (**d**) key-points for variation of weld width *H* and crack length *a*; (**e**) detail of finite element mesh of model with heterogeneous weld.

Due to the possibility of crack formation in the original and the repaired part of the weld, both variants were analyzed. Due to geometry and load symmetry, a plane strain two-dimensional numerical model of one half of an SE(B) specimen with homogeneous weld metal (WM) was created in ANSYS [27] (Figure 2a,c). The model was verified comparing finite element results with the analytical method of slip line field analysis [17] that is used in the analytical analysis of strength mismatch welds. Results of verification showed very good agreement between the results of numerical and slip line field analyses, and this verification is already published in [21]. A single change was made to the verified numerical model, in the form of splitting the homogeneous weld into two equal portions of over-matched and under-matched weld metal to form a heterogeneous weld (Figure 2b,e).

To determine the influence of weld width *H* and crack length *a* on the yield load, the width of the weld *H* was varied as *H* = *W*/2, *H* = *W*/4, *H* = *W*/8, *H* = *W*/16 and *H* = *W*/24, while the crack length in relation to the height of the specimen *W* was varied as *a*/*W* = 0.1, *a*/*W* = 0.2, *a*/*W* = 0.3, *a*/*W* = 0.4 and *a*/*W* = 0.5 (Figure 2d). The length of the specimen *S* was kept constant. 

The base metal (BM) and the weld metals (OM and UM) were modeled as isotropic linearly elastic and nearly ideally plastic materials with Poisson’s ratio of 0.3 and with a Young’s modulus of 202 GPa for base metal, 200 GPa for over-matched and 206 GPa for under-matched metal. Elasticity mismatch also have an influence on the fracture behavior of a welded joint [28,29], but this slight degree of elasticity mismatch did not show an influence on the values of the obtained yield loads compared to ones obtained without elasticity mismatch. The yield strength of the base metal was 545 MPa. The strength mismatch of base and weld metals are varied on three levels: over-match metal with mismatch factor *M*_OM_ = 2, 1.5 and 1.19 and under-match metal with mismatch factor *M*_UM_ = 0.86, 0.75 and 0.5. Yield strength and mismatch factors *M*_UM_ = 0.86 and *M*_OM_ = 1.19 were chosen due to later comparison to experimental results.

Due to the faster convergence of the results, a practically negligible strain hardening exponent was used, which did not affect the results but significantly reduced the computation time. To avoid the incompressibility problem, an isoparametric planar element with eight nodes, plane strain and reduced integration was used. Singular elements with a size of 100 µm were used in the first ring of elements around the crack tip to produce the square root singularity of the stress–strain field. Models were meshed with 1847 finite elements and with 5690 nodes. Prepared models were loaded with a load large enough to cause the material to yield through the entire cross-section of the model. 

The load was increased gradually in small increments to accurately determine the load of plasticization of the entire net section of the specimen, indicating the formation of a plastic hinge and plastic collapse. As a criterion for material flow, the von Mises criterion was used. A total of 450 simulations were performed for a crack located in an over-matched and under-matched part of the weld.

## 4. Results of Finite Element Analysis

**Figure 3:**
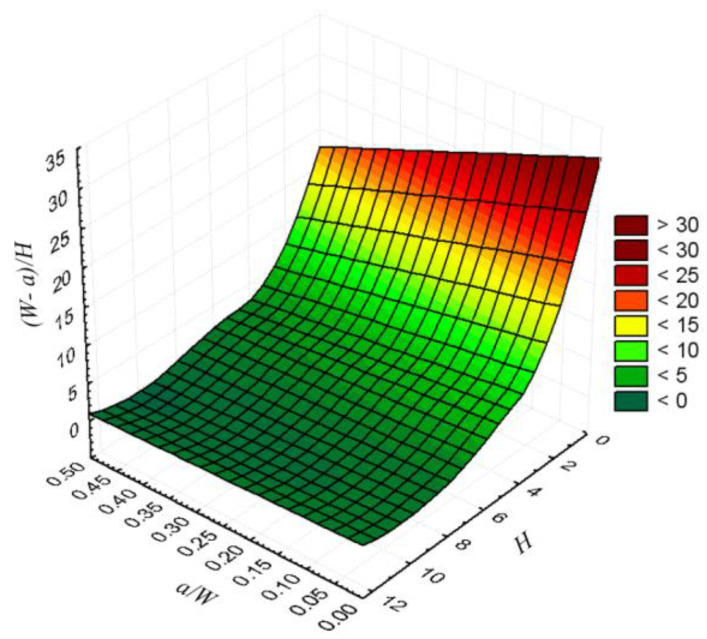
Dependence of weld slenderness (*W*−*a*)/*H* on weld width *H* and crack length *a/W*.

Obtained yield loads for heterogeneous weld were normalized with yield loads of all base specimen according to [17] and presented in diagrams depending on the weld slenderness (*W* − *a*)/*H*. To facilitate the interpretation of the results, the dependence of weld slenderness on crack length and weld width is shown in Figure 3. It can be seen that the slenderness of the weld increases significantly with decreasing weld width and becomes less pronounced with decreasing crack length.

### 4.1. Yield Load Solutions for a Crack in the Over-Matched Part of the Weld

**Figure 4:**
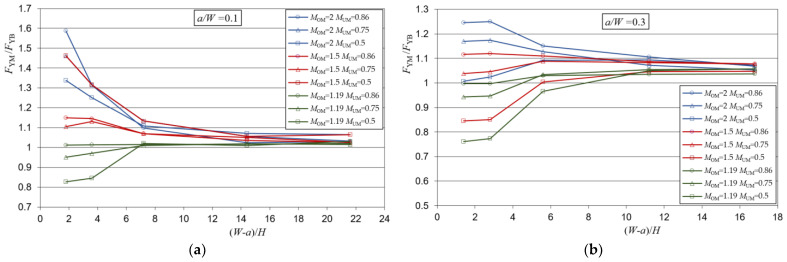

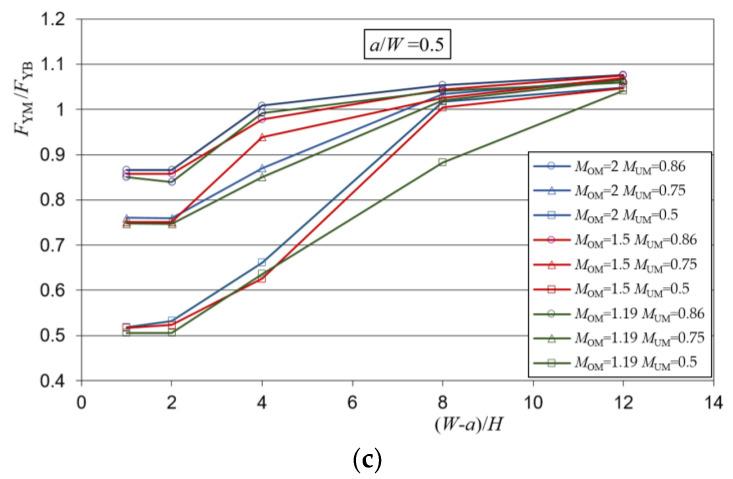
Mismatch yield loads for the heterogeneous weld and for a crack in the over-matched part of the weld: (**a**) shallow crack—*a*/*W* = 0.1; (**b**) medium length crack—*a*/*W* = 0.3; (**c**) deep crack—*a*/*W* = 0.5.

Yield load solutions as a result of the analysis of a heterogeneous weld with a crack in the over-matched part of the weld in the function of weld slenderness (*W* − *a*)/*H* are presented in Figure 4. 

From Figure 4, it can be seen that the dispersion of the yield load solutions at lower weld slenderness depends on the present weld metals, while at higher weld slenderness, the solutions of all metal combinations and all crack lengths approach an asymptotic value. This value is slightly higher than the value 1, indicating a slight increase in the strength of the weld compared to the component of the homogeneous base metal.

When the slenderness of the weld is lower, different effects occur depending on the length of the crack in the weld. For the crack *a*/*W* = 0.5, there is only under-matched metal in front of the crack, which is represented by the mismatch factor *M*_UM_. Therefore, the solutions were the values 0.5, 0.75 and 0.86 because the dominant metal is in front of the crack. Although the solutions were these values, it is noticeable that they are actually slightly larger, which is a consequence of the formation of the yield zone partially through the over-matched metal too, which has a higher value of the mismatch factor *M*_OM_. 

**Figure 5:**
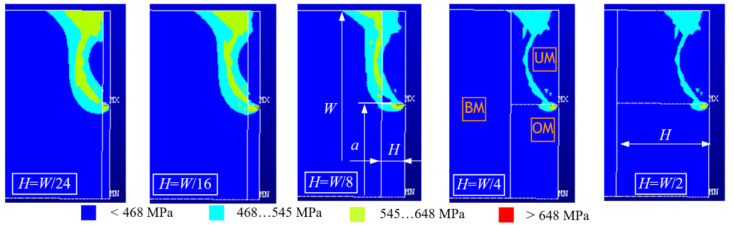
Formation of the yield zone in a heterogeneous weld with a crack in the over-matched part of the weld for varying weld width *H* and constant crack length *a*/*W* = 0.5.

Figure 5 shows formation of the yield zone in a heterogeneous weld with a crack in the over-matched part of the weld for varying weld width *H* and constant crack length *a*/*W* = 0.5 for mismatch factors *M*_OM_ = 1.19 and *M*_UM_ = 0.86.

The appearance of the yield zones is similar for all geometries, and depending on the width of the weld, the yield zone extends through two or all three materials. For narrow weld widths *H* = *W*/24, *H* = *W*/16 and *H* = *W*/8, the material yield zone spreads through the base metal and both weld metals, while for wider welds *H* = *W*/4 and *H* = *W*/2, the yield zone stays within the weld metal.

As the size of the crack decreases, the metal in which the crack is located becomes more influential. This is particularly pronounced for combinations of metals whose mismatch factors *M*_OM_ and *M*_UM_ differ significantly, while the solutions for combinations of metals with closer values of *M*_OM_ and *M*_UM_ approach the values of 1 of the base metal. For example, for the combination of metals *M*_OM_ = 2 and *M*_UM_ = 0.5, the solutions range from 0.5 to 1.6, and for the combination of *M*_OM_ = 1.19 and *M*_UM_ = 0.86, the solutions are almost everywhere uniform and closer to the value 1.

The results of the numerical analyses for a crack in the over-matched part of the weld were processed in the software package TuringBot [30] using a symbolic regression algorithm to derive mathematical formulas from numerically obtained values with high efficiency. An equation that estimates the values of the ratio of the yield loads for the heterogeneous weld and the whole base metal was obtained. A high goodness-of-fit of the selected model was confirmed with the R-squared value 0.938 and RMS error 0.03748. The equation considers values of the over-match strength mismatch *M*_OM_, under-match strength mismatch *M*_UM_, the weld width *H* and the crack length *a*/*W*:

(2) $$\frac{F_{\mathrm{YM}}}{F_{\mathrm{YB}}}=1-\frac{1+H\left[\left(M_{\mathrm{UM}}-M_{\mathrm{OM}}\frac{a}{W}\right)\left(1+2\frac{a}{W}-\frac{1}{H}\right)+M_{\mathrm{OM}}-2\right]}{-20-\left(\frac{H}{3}+3\right)\left(H-10\right)\frac{a}{W}}$$

### 4.2. Yield Load Solutions for a Crack in the Under-Matched Part of the Weld

**Figure 6:**
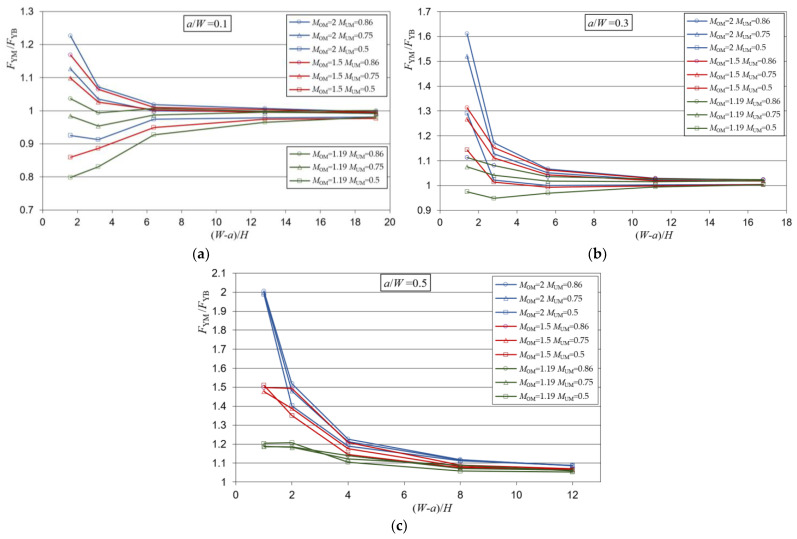
Mismatch yield loads for the heterogeneous weld and for a crack in the under-matched part of the weld: (**a**) shallow crack—*a*/*W* = 0.1; (**b**) medium length crack—*a*/*W* = 0.3; (**c**) deep crack—*a*/*W* = 0.5.

Yield load solutions as a result of the analysis of a heterogeneous weld with a crack in the under-matched part of the weld in the function of weld slenderness (*W* − *a*)/*H* are presented in Figure 6.

Results of the analysis, shown in Figure 6, indicated that for lower weld slenderness, the yield load solutions differ depending on the weld metals present in the weld, while for higher weld slenderness, the yield solutions of all metal combinations, as well as for all crack lengths, approached the value 1.

When the weld was less slender, the weld showed different behavior depending on the length of the crack in the weld. For the crack *a*/*W* = 0.5, only the OM was in front of the crack, therefore the yield loads were the values 1.19, 1.5 and 2. This happens because the metal in front of the crack, which has the over-match characteristic, takes the dominant role. Although the solutions were these values, it can be noted that they were somewhat lower, which was a consequence of the partial propagation of the yield zone also through the under-matched metal. 

As the length of the crack decreases, the metal in which the crack is located also becomes more influential. Similar to the case where the crack was in an over-matched metal, it can be observed that the solutions with closer values of *M*_OM_ and *M*_UM_ approached the values of 1 of the base metal. For example, for the combination of *M*_OM_ = 2 and *M*_UM_ = 0.5, the solutions ranged from 0.9 to 2, and for the combination of *M*_OM_ = 1.19 and *M*_UM_ = 0.86, the solutions were almost uniform and were everywhere closer to the value of 1.

The results of the analysis for a crack in the under-matched part of the weld were also processed in the software package TuringBot using a symbolic regression algorithm and an equation for the estimation of the values of the ratio of yield loads for the heterogeneous weld and the whole base metal was obtained:

(3) $$\frac{F_{\mathrm{YM}}}{F_{\mathrm{YB}}}=\frac{H-\left[H\cdot M_{\mathrm{OM}}\left(M_{\mathrm{UM}}+\frac{a}{W}\right)-\left(M_{\mathrm{OM}}-3\frac{a}{W}\right)\left(M_{\mathrm{OM}}-\frac{1}{H}\right)\right]}{\frac{23}{M_{\mathrm{UM}}}\frac{a}{W}-34}+1$$

A high goodness-of-fit for model with a crack in the under-matched part of the weld was confirmed with the R-squared value 0.953 and RMS error 0.04095. 

## 5. Experimental Investigation

**Figure 7:**
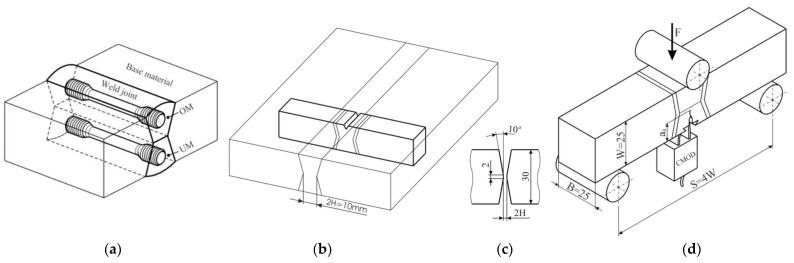
Tensile and fracture toughness specimens: (**a**) position and orientation of tensile specimens; (**b**) fracture toughness specimen notch orientation; (**c**) weld arrangement; (**d**) three point bending specimen SE(B) for fracture toughness testing.

For this investigation, standard SE(B) test specimens were prepared from the welded plate. For the base metal (BM), NIOMOL 490 was used as a high-strength, low-alloy, fine-grain steel in the hardened and tempered condition according to the HT 50 grade. Using the flux cord arc welding procedure and two tubular wires as filler material FILTUB 75 and VAC 60 as an over-match and under-match material, a heterogeneous weld was produced with the strength mismatch factor 1.19 and 0.86. Mechanical properties of the base metal and OM and UM part of the weld, shown in Table 1, were obtained by a tensile test. Five round specimens with a 5 mm diameter were used for each metal. The position and orientation of round specimens in the weld joint are shown in Figure 7a. The chemical composition of BM, UM and OM metal, shown in Table 2, is provided by the manufacturer, where OM and UM chemical composition is provided for pure weld metal.

For fracture toughness testing, specimens were prepared, and a single-sample method was used according to the standard BS 7448 [31]. CTOD fracture toughness specimens with dimensions and notch orientation are shown on Figure 7. The CTOD tests were carried out at room temperature (+24 °C) under displacement control (1 mm/min). Load *F*, total displacement, crack tip (CTOD) and crack mouth opening displacement (CMOD) were recorded during the tests. Tests were performed for two configurations: with a crack in the over-matched part of the weld and with a crack in the under-matched part of the weld. A total of 14 specimens were tested: 7 specimens with a crack initiated in the OM part of the weld and 7 with a crack initiated in the UM part of the weld. 

**Figure 8:**
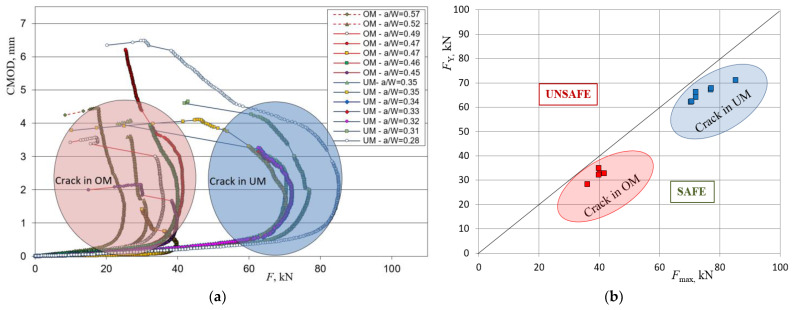
Experimentally obtained results: (**a**) loading curves for specimens with a crack in the OM and UM part of the weld; (**b**) comparison of experimentally obtained maximum load with a yield load obtained by numerical analysis for a crack in the OM and UM part of the weld.

The plots of load versus CMOD were obtained and shown in Figure 8a. During fatigue pre-cracking in two specimens, with a notch in the OM part of the weld, a crack reached the fusion line between the OM and UM and advanced to the UM part of the weld (Figure 8a shown with dotted lines); therefore, they are omitted in later comparison with the yield load solutions for a heterogeneous weld. For every sample, a crack location (OM or UM) and initial crack length compared to the height of the specimen (*a*/*W*) is shown in the legend. 

From the loading curve plots, it can be seen that every specimen for each configuration shows a certain period of stable crack propagation and reaches a maximum load, followed by a load decrease and unstable crack propagation. The difference in slopes in the diagrams and values of maximum load were due to different initial crack lengths and position of the crack (OM or UM part of the weld), and a separation of curves for the crack located in the OM part of the weld and advancing to the UM and vice versa can be noted. 

The maximum load was determined for each specimen and compared with the yield load solutions obtained by expressions (2) and (3). This comparison is presented in Figure 8b with a yield load versus maximum load plot. Grouping of results is noted for all specimens with a crack in the OM part of the weld as well as with a crack in the UM part of the weld. This was due to relatively similar crack lengths and location of the crack either in the OM or in the UM part of the weld. Yield loads generated from numerically obtained mathematical models for the crack located in the OM and UM part of the weld were lower and conservative enough compared to experimental results. However, it is likely that even less conservative results could be obtained if yield load solutions were implemented as input parameters for evaluating the welded component using structural integrity assessment procedures.

## 6. Conclusions

As a result of a systematic numerical study, the yield load solutions for SE(B) specimens with a heterogeneous I-shaped weld with an equal share of over-matched and under-matched metal in a welded joint were obtained. Comparing the numerically obtained results in terms of crack position, it can be concluded that a heterogeneous welded joint with a crack in the under-matched metal shows a higher loading capacity than a welded joint with a crack in the over-matched part of a weld. This indicates that in the context of yield load, the metal in front of the crack has a greater effect on the fracture resistance than the metal in which the crack is located. This effect is more pronounced for welds with lower values of weld slenderness (*W* − *a*)/*H*. After processing of the numerically obtained results using a symbolic regression algorithm, solutions for the yield loads were proposed with two models: for the crack in the under-matched and for the crack in the over-matched part of the weld. The models were validated with experimental results and they provided sufficiently conservative results compared to the experiment.

## Figures and Tables

**Table 1:** Mechanical properties of base and weld metals with mismatch factor.

Material	*R*_p0.2_, MPa	*R*_m_, MPa	*E,* GPa	*M*
Base metal (NIOMOL 490)	545	648	202	-
Over-matched (FILTUB 75)	648	744	184	1.19
Under-matched (VAC 60)	468	590	206	0.86

**Table 2:** Chemical composition of base and weld metals.

Material	C	Si	Mn	P	S	Cr	Mo	Ni
Base metal (NIOMOL 490)	0.123	0.33	0.56	0.003	0.002	0.57	0.34	0.13
Over-matched (FILTUB 75)	0.040	0.16	0.95	0.011	0.021	0.49	0.42	2.06
Under-matched (VAC 60)	0.096	0.58	1.24	0.013	0.160	0.07	0.02	0.03

## Data Availability

Not applicable.
